# Evaluation of Antipsychotic Drugs’ Stability in Oral Fluid Samples

**DOI:** 10.3390/molecules28052030

**Published:** 2023-02-21

**Authors:** Carina Gameiro, Joana Gonçalves, Sofia Soares, Tiago Rosado, André R. T. S. Araujo, Luís A. Passarinha, Mário Barroso, Eugenia Gallardo

**Affiliations:** 1 Centro de Investigação em Ciências da Saúde (CICS-UBI), Universidade da Beira Interior, Av. Infante D. Henrique, 6200-506 Covilhã, Portugal; 2 Laboratório de Fármaco-Toxicologia, UBIMedical, Universidade da Beira Interior, Estrada Municipal 506, 6200-284 Covilhã, Portugal; 3 Unidade de Investigação para o Desenvolvimento do Interior, Instituto Politécnico da Guarda, Avenida Dr. Francisco de Sá Carneiro, No. 50, 6300-559 Guarda, Portugal; 4 LAQV, REQUIMTE, Laboratory of Applied Chemistry, Department of Chemical Sciences, Faculty of Pharmacy, Porto University, Rua Jorge Viterbo Ferreira, No. 228, 4050-313 Porto, Portugal; 5 UCIBIO—Applied Molecular Biosciences Unit, Departamento de Química, Faculdade de Ciências e Tecnologia, Universidade NOVA de Lisboa, 2829-516 Caparica, Portugal; 6 Associate Laboratory i4HB—Institute for Health and Bioeconomy, NOVA School of Science and Technology, Universidade NOVA, 2819-516 Caparica, Portugal; 7 Serviço de Química e Toxicologia Forenses, Instituto de Medicina Legal e Ciências Forenses—Delegação do Sul, 1169-201 Lisboa, Portugal

**Keywords:** antipsychotics, oral fluid, dried saliva spots, GC-MS/MS, stability

## Abstract

Antipsychotics have narrow therapeutic windows, and their monitoring in biological fluids is therefore important; consequently, stability in those fluids must be investigated during method development and validation. This work evaluates the stability of chlorpromazine, levomepromazine, cyamemazine, clozapine, haloperidol, and quetiapine in oral fluid (OF) samples, using the dried saliva spots (DSS) sampling approach and gas chromatography coupled to tandem mass spectrometry. Since many parameters can influence the stability of the target analytes, design of experiments was adopted to check the crucial factors that affect that stability in a multivariate fashion. The studied parameters were the presence of preservatives at different concentrations, temperature, light, and time. It was possible to observe that antipsychotic stability improved when OF samples in DSS were stored at 4 °C, with a low ascorbic acid concentration, and in the absence of light. With these conditions, chlorpromazine and quetiapine were stable for 14 days, clozapine and haloperidol were stable for 28 days, levomepromazine remained stable for 44 days, and cyamemazine was stable for the entire monitored period (146 days). This is the first study that evaluates the stability of these antipsychotics in OF samples after application to DSS cards.

## 1. Introduction

Schizophrenia is considered one of the ten main causes of disability around the world, affecting around 1% of the population [1]. The increase in suicides related to this psychological disorder has generated great concern, since 10% of the attempts are successful [2]. Antipsychotics are the main drugs used for the treatment of schizophrenia, among other psychiatric illnesses, with the aim of reducing some of its symptoms, as well as preventing relapses [3]. These drugs can be classified into first-generation or second-generation antipsychotics, with the former acting mainly on the blockage of D2 receptors, while the latter acts mainly on the blockage of the 5-HT2A serotonergic receptors, which explain their difference to control the characteristic positive and negative symptoms of this illness [4].

In forensic toxicology, blood and urine are the most commonly used samples for both ante- and post-mortem analysis. However, the blood collection method is considered invasive, and urine can be easily adulterated; in addition, some substances, such as highly lipophilic compounds, may be difficult to detect [5,6]. Oral fluid (OF) has been gaining interest as an alternative matrix, particularly in analytical chemistry and forensic toxicology laboratories [7,8]. This sample has the advantages of being collected in an easy, fast, and non-invasive way, being easy to manipulate, transport and store, revealing a low risk of tampering with, and presenting the parent drugs in higher concentrations [7,9,10,11]. However, a reduced sample volume might be available and analytes may be present in low concentrations [7,9,10]. Additionally, substances administered orally are usually found in higher concentrations after consumption, due to residual substance left in the oral cavity, compromising the accuracy of the results; hence, their concentration may not properly reflect blood concentration [7].

In recent years, a number of analytical methods have been developed and reported, using the dried saliva spots (DSS) approach, together with analyses by liquid chromatography coupled to tandem mass spectrometry (LC-MS/MS) [12], high performance liquid chromatography coupled to diode array detector (HPLC-DAD) [13], liquid chromatography coupled to mass spectrometry (LC-MS) [14], and gas chromatography coupled to tandem mass spectrometry (GC-MS/MS) [1,15,16]. DSS is a sampling technique in which OF samples are spotted on filter paper, and the target analytes are then extracted with an organic solvent [12]. This approach has become very promising regarding metabolic, congenital, and hereditary disorders diagnosis and therapeutic drug monitoring [17].

Due to bureaucratic issues or laboratorial work volume, it is not always possible to analyse samples as soon as they arrive to the laboratory [18]. Sample analysis may be delayed for days, weeks, or even months since the time of collection [18]. Therefore, knowing and evaluating the stability of analytes in different biological specimens is essential for a better understanding of the obtained results, in a reliable manner [19]. The stability of drugs in bioanalytical procedures is mostly investigated by using a short-term evaluation and freeze/thaw study. Usually, these data are obtained within the analytical method validation stage, and are often unclear concerning different storage conditions and longer periods of storage [20]. 

We previously developed and validated an analytic method to determine the antipsychotics chlorpromazine, levomepromazine, cyamemazine, clozapine, haloperidol, and quetiapine using DSS and GC-MS/MS [1]. In the present work, the same analytical method was used to investigate the stability of these antipsychotics. Different preservatives were added to the OF samples and different storage conditions of the DSS were evaluated by means of the design of experiments (DOE) analysis. The study was subsequently carried out for a period of 146 days with the optimal storage conditions.

To the best of our knowledge, this is first work that evaluates the stability of these antipsychotics in OF samples using the DSS technique and GC-MS/MS.

## 2. Results and Discussion

### 2.1. Optimization of the Stability Protocol

In the present study, the storage temperature, the presence or absence of light, the concentration of the three preservatives (ascorbic acid, sodium fluoride, and sodium azide) tested, and the storage time of the DSS were evaluated and optimized. Through the response of each analyte, obtained from the ratio of the absolute area of analytes and IS, it was possible to obtain pareto graphs, main effects plots, and interactions plots of each studied factor for each compound. The pareto graphs present the parameter or parameters with greatest influence on the stability of the target analytes (by the length of the bar), while the main effects plot examines differences between levels (e.g., low or high concentration) for one or more factors in the study. The effect is calculated based on the relative peak area resultant of the GC-MS/MS analysis.

**Figure 1:**
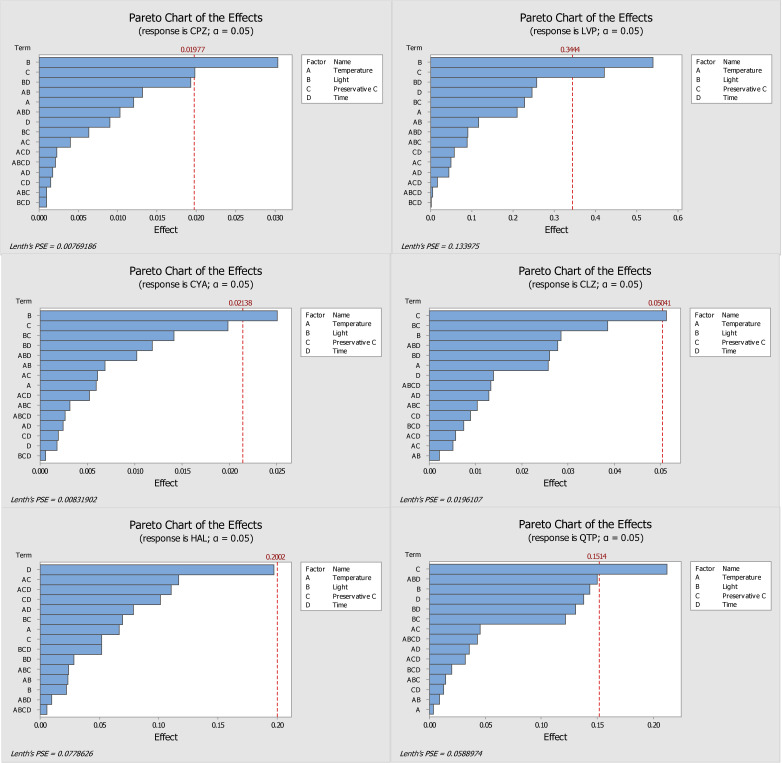
Pareto diagrams of the compounds under study (CPZ—chlorpromazine, LVP—levomepromazine, CYA—cyamemazine, CLZ—clozapine, HAL—haloperidol, QTP—quetiapine) obtained with ascorbic acid as a preservative. The bars represent the effects of factors or a combination of factors: A—Temperature, B—Light, C—Preservative, and D—Time.

**Figure 2:**
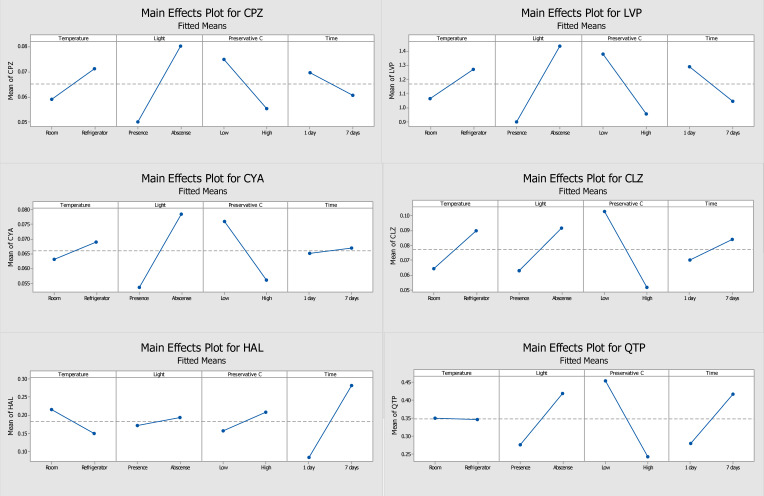
Main effects plots from ascorbic acid used as the preservative, which demonstrates the main factors in each parameter of each analyte (CPZ—chlorpromazine, LVP—levomepromazine, CYA—cyamemazine, CLZ—clozapine, HAL—haloperidol, QTP—quetiapine). The slope of the lines represents the effect of the response for the factors under study: temperature (25 °C or 4 °C), light (presence or absence), preservative concentration level (low or high), and storage time (1 or 7 days).

**Figure 3:**
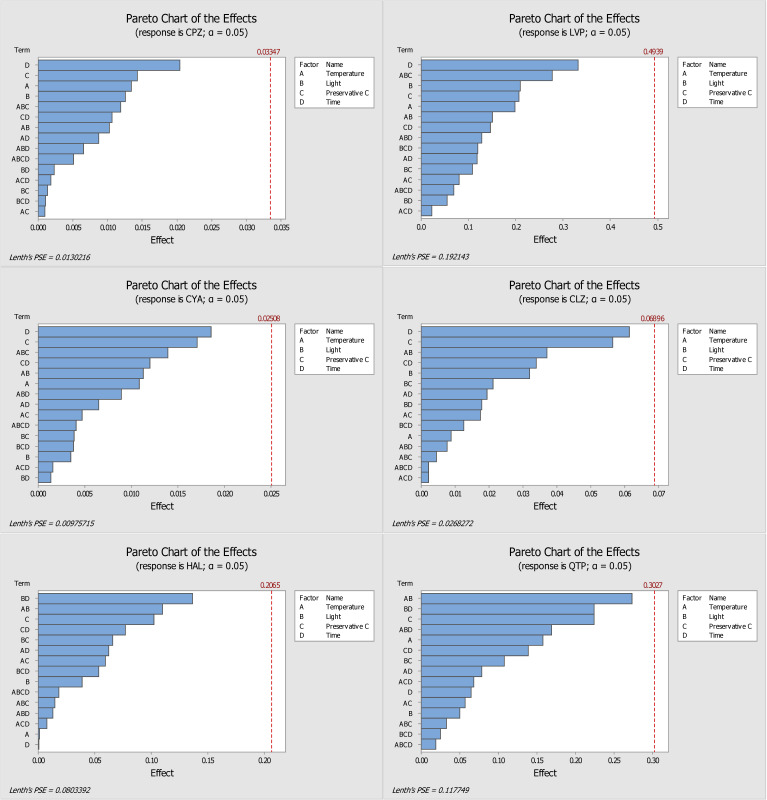
Pareto diagrams of the compounds under study (CPZ—chlorpromazine, LVP—levomepromazine, CYA—cyamemazine, CLZ—clozapine, HAL—haloperidol, QTP—quetiapine) obtained with sodium fluoride as preservative. The bars represent the effects of factors or a combination of factors: A—Temperature, B—Light, C—Preservative, and D—Time.

**Figure 4:**
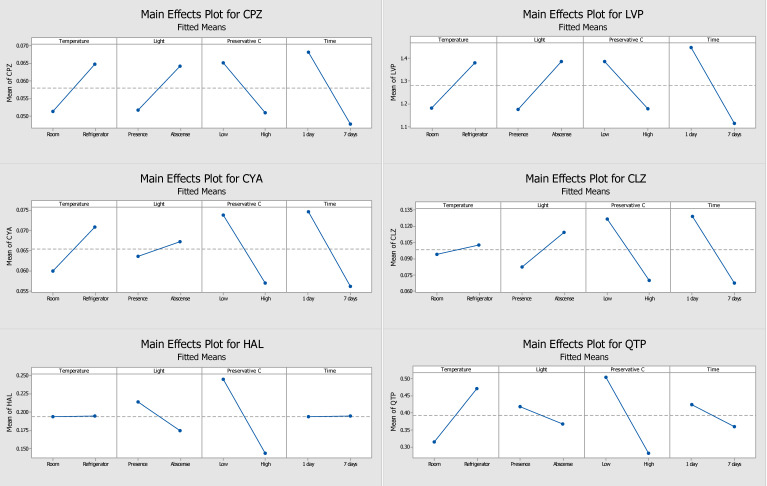
Main effects plots from sodium fluoride used as the preservative, which demonstrates the main factors in each parameter of each analyte (CPZ—chlorpromazine, LVP—levomepromazine, CYA—cyamemazine, CLZ—clozapine, HAL—haloperidol, QTP—quetiapine). The slope of the lines represents the effect of the response for the factors under study: temperature (25 °C or 4 °C), light (presence or absence), preservative concentration level (low or high), and storage time (1 or 7 days).

Figure 1 and Figure 2 show the pareto graphs and the main effects plots, respectively, obtained for each target analyte when ascorbic acid was tested as a preservative. Figure 3 and Figure 4 present the pareto graphs and the main effects plots, respectively, obtained for each target analyte when sodium fluoride was tested as a preservative.

Analysis of the pareto diagram (Figure 1) reveals a significant influence on the response to at least one evaluated factor, except for haloperidol. The factors that had a significant influence on the response (relative peak area) were the presence of light (for chlorpromazine, levomepromazine, and cyamemazine) and the concentration of ascorbic acid (for chlorpromazine, levomepromazine, clozapine, and quetiapine). Haloperidol was the only analyte that did not appear to suffer a significant influence by the studied variables; however, the storage time of the DSS was the parameter with greatest influence. Regarding the main effects plots (Figure 2), it was possible to determine the best storage conditions (level of each variable) for the analytes. Four of the six analytes (chlorpromazine, levomepromazine, clozapine, and cyamemazine) had a greater response when the DSS were stored at 4 °C, while the other two analytes (quetiapine and haloperidol) presented a better response when the DSS were stored at room temperature. All analytes had a greater response when the DSS were stored in the absence of light and in the presence of a low concentration of ascorbic acid. It is important to highlight that these two variables were the only ones that had a significant influence in most of the studied antipsychotics. Only haloperidol revealed a greater response when the DSS were stored with high concentrations of ascorbic acid. Lastly, by using ascorbic acid as preservative, the storage time appeared to have no significant influence for any of the analytes, with only two of them (chlorpromazine and levomepromazine) revealing a greater response after one day of storage. Overall, it was found that the analytes present in the DSS, when stored at 4 °C, in the absence of light and with a low concentration of ascorbic acid, had better responses, which means that their stability was higher.

Regarding sodium fluoride preservative, and by analysing the pareto charts (Figure 3), it is possible to conclude that none of the studied variables had a significant influence in the response. Despite this, there are some factors that affect the results more than others, namely the storage time for chlorpromazine, levomepromazine, cyamemazine, and clozapine, the combination of storage time and the influence of light, in the case of haloperidol, and the combination of storage temperature with the influence of light for quetiapine. The main effects plots (Figure 4) show that all analytes had a better response when stored at 4 °C. Additionally, four analytes (chlorpromazine, levomepromazine, clozapine, and cyamemazine) revealed a greater response when stored in the absence of light, while for quetiapine and haloperidol, the improvement is observed in the presence of light. For all analytes, and as proven for ascorbic acid, storage with a low concentration of sodium fluoride provided the best response. Concerning the storage time for chlorpromazine, levomepromazine, clozapine, cyamemazine, and quetiapine, the response was greater on the first day, while the opposite happened for haloperidol. Based on these interpretations, it is possible to deduce that, after 7 days, high losses of analytes occur, so sodium fluoride does not appear to be a good preservative option regarding these compounds.

**Figure 5:**
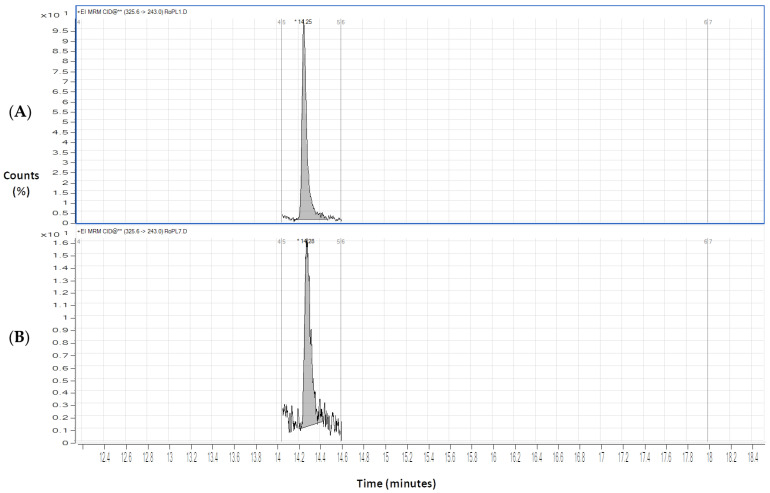
Chromatograms on the first (**A**) and seventh (**B**) days of extraction for clozapine (CLZ) (200 ng/mL) with sodium azide (0.1%).

Finally, sodium azide was also tested as a preservative in our DSS samples, and stored under the same conditions used for ascorbic acid and sodium fluoride. Nonetheless, the chromatograms of all analytes, after 7 days of storage, revealed a high level of degradation; hence, it was concluded to be the least suitable preservative. Figure 5 shows, as example, the obtained chromatogram of clozapine after one day (A) and seven days (B) of the DSS storage with sodium azide. After 7 days (B), clozapine could not be quantified.

### 2.2. DOE Performed without Preservative

After the stability study for 7 days with the selected preservatives, a DOE was performed without preservatives added (also for 7 days). This assay was performed only with OF fortified with the same antipsychotic drugs and applied to DSS, as previously described. The aim of this assay was to observe the analytes behavior in the absence of the preservative after the first and seventh day of storage in the different storage conditions.

The response of the compounds was studied by calculating the means of the values obtained between the first and seventh day (Table 1). The response was measured by the relative peak area (peak area of the analyte/peak area of IS) obtained for each of the target antipsychotic drugs.

From Table 1, it is possible to observe the compounds’ behaviour when stored at 4 °C and 25 °C; likewise in the presence and absence of light. The results obtained for the light and temperature effects are mostly in agreement with the results obtained in the previous assay performed with preservative.

It is possible to conclude that, in terms of temperature, for chlorpromazine and levomepromazine, the response is higher when stored at 4 °C; on the other hand, for cyamemazine, clozapine, and quetiapine, the response obtained in storage at 4 °C and 25 °C is quite similar. Regarding haloperidol, the best response obtained was with storage at 25 °C. In the case of the influence of light, for all analytes, the best responses obtained were in its absence (conditions B and C).

### 2.3. Final Conditions for Long-Term Stability Assay

The best storage conditions for the DSS were chosen by the integration of all the obtained diagrams (pareto and main effects plots for ascorbic acid and sodium fluoride); likewise the DOE performed without preservative.

Evaluating the ascorbic acid (Figure 1) and sodium fluoride (Figure 3) pareto charts, it is possible to observe that, for ascorbic acid, there is at least a significant factor that influences the response obtained for all analytes, except for haloperidol. On the other hand, when sodium fluoride was used, no factor appeared to influence significantly the obtained responses. In the case of storage with ascorbic acid, it is possible to observe that the presence of light and preservative concentration affected the analytes chlorpromazine, levomepromazine, and cyamemazine, and chlorpromazine, levomepromazine, clozapine, and quetiapine, respectively. Although it is also noted that these factors are also the predominant in the responses obtained with sodium fluoride, these have no statistical significance in the latter. Comparing the results presented in the tables of these two preservatives, smaller losses of the target analytes were observed when the DSS are stored with ascorbic acid after 7 days, when compared to those with sodium fluoride. 

Thus, ascorbic acid was elected the best preservative and chosen for the long-term stability assay. For ascorbic acid, a greater response had been observed when the DSS were stored at 4 °C, in the absence of light and with a low concentration of preservative; hence, these were adopted as optimal conditions (Table 2).

Furthermore, the DOE responses without preservative were compared with the DOE responses at low ascorbic acid concentration (both at the defined conditions of light and temperature). The response was, once again, measured by the relative peak area. According to the obtained results, no differences were observed on results with and without preservative for chlorpromazine, levomepromazine, and cyamemazine, while for clozapine, haloperidol, and quetiapine, the presence of ascorbic acid improved the response of these analytes.

### 2.4. Samples Stability

Long-term stability was studied for 146 days. This period was divided into nine assays, so stability was studied at the day of sample preparation (day 1) and on days 3, 7, 14, 21, 28, 44, 117, and 146. 

For day 1, a percentage of 100% was established for the concentration of the detected analyte. The following quantifications were made considering the concentration of day 1. For each day of analysis, DSS were prepared in triplicate. All coefficients of variation (CV) between assays were within ± 20%. Analyte instability was defined as a deviation of more than 20% from the initial concentration. Table 3 shows the obtained results in this assay.

**Figure 6:**
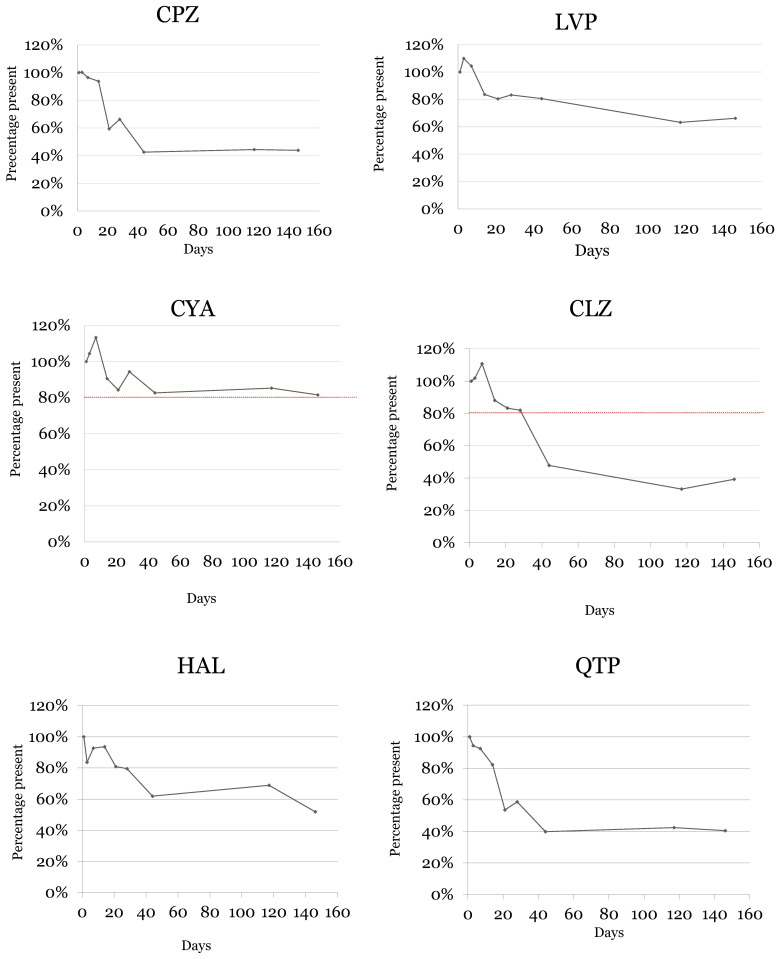
Stability of antipsychotics on DSS over 146 days in optimal conditions (low concentration of ascorbic acid, absence of light, and 4 °C). CPZ—chlorpromazine, LVP—levomepromazine, CYA—cyamemazine, CLZ—clozapine, HAL—haloperidol, QTP—quetiapine.

A significant decrease was observed for chlorpromazine (Figure 6 and Table 3) after 14 days, which means that chlorpromazine was stable (with a degradation lower than 20%) until 14 days under the optimized conditions. After the 44 days of storage, this analyte showed losses above 50%.

In the study conducted by Caramelo et al. [1], the storage of DSS with chlorpromazine for 4 days at 25 °C in the presence of light and without any preservative revealed deviations of its initial concentration of 8.7 and 8.4%. The optimized storage conditions in the present work resulted in no losses of chlorpromazine for 3 days. After 7 days of storage under these conditions, chlorpromazine showed a 4% deviation from the first day, whereas in the study by Caramelo et al. [1], the compound was no longer stable by the eighth day. 

Regarding levomepromazine, a stability of 44 days was assured (Figure 6 and Table 3), with a slight decrease on the concentration between 44 and 117 days of storage at the optimized conditions. 

In the study carried out by Caramelo et al. [1], levomepromazine showed variations up to 11.1% after 4 days of storage. The herein described work revealed variations from its initial concentration of 10% after 3 days of storage. After 7 days of storage had elapsed, this analyte still remained stable, while, in the aforementioned work, with 8 days of storage, this analyte revealed losses above 50% [1]. Hence, with the optimized conditions in the present study, the stability of levomepromazine improved after 7 days. According to Figure 6, this analyte has a better response at 4 °C and in the absence of light (significant condition); therefore, when stored under defined conditions for long-term study, the results obtained would be expected. 

Concerning cyamemazine, this compound was stable over the whole monitored period (Figure 6 and Table 3). 

According to Caramelo et al. [1], variations from initial concentration up to 10.9% were obtained with 4 days of storage. After 8 days of storage, cyamemazine was no longer stable. In the present work, the stability improved greatly at defined conditions: once after 3 days of storage, the initial concentration only changed by 2%; and after 146 days, the losses were lower than 20%. These results prove that cyamemazine can be quantified with accuracy after more than 4 months in OF stored in DSS under the optimized conditions. 

As for clozapine, this compound remained stable for 28 days in the defined storage conditions, revealing a huge decrease in its concentration between day 28 and day 44 of storage (Figure 6 and Table 3). 

In the absence of preservative and stored in DSS with the presence of light at 25 °C, clozapine revealed deviations from the initial concentration of up to 1.2% after 4 days. After the eighth day of storage, losses greater than 50% were observed [1]. On the other hand, in the present study, when clozapine is stored with ascorbic acid at 4 °C in the absence of light, significant losses are only observed after 7–8 days of storage. In a study carried out by Fisher et al. [21], the stability of clozapine was evaluated in neat OF. The authors reported that this drug remained stable for 1 week if OF samples were stored at temperatures between 2 and 8 °C. In another study carried out by Patteet et al. [22], clozapine present in OF collected with the Quantisal™ device and stored in a polypropylene tube at 4 °C remained stable for 7 days.

Regarding haloperidol, this drug revealed good stability until the 28th day of storage (Figure 6 and Table 3), with degradation of more than 30% after 44 days of storage at the optimized conditions in the herein described work. 

According to Caramelo et al. [1], haloperidol revealed slight deviations from its initial concentration after 4 days of storage on DSS without preservative, at 25 °C with the presence of light. After 8 days of storage, this drug remained stable. In the present work, the losses in the 4th and 8th day were between 7 and 16%. Although haloperidol was still stable, it seems that the losses observed in the present work were slightly greater than those reported by Caramelo et al. [1]. The latter can be explained by the fact that in this study DSS were not stored at 25 °C, the temperature that gives the best response to haloperidol according to Figure 2. Nonetheless, this drug revealed good stability in OF stored under the defined conditions. According to Patteet et al. [22], haloperidol present in OF collected with the Quantisal™ device and stored in a polypropylene tube at 4 °C remained stable for 7 days. 

Finally, quetiapine was stable for 14 days (Figure 6 and Table 3). After this period of time, quetiapine revealed losses greater than 40% at the defined storage conditions.

According to Caramelo et al. [1], quetiapine showed deviations from its initial concentration up to 6.1% when stored for 4 days, and up to 12.7% when stored for 8 days. In the present study, losses of 6% and 7% were observed after 3 and 7 days, respectively. Compared with Caramelo et al. [1], the herein described study revealed that the stability of quetiapine was the same after 3–4 days, while after one week there were great improvements if the optimized storage conditions were adopted. In the work carried out by Fisher et al. [21], quetiapine remained stable for 1 week when stored at temperatures of 2 to 8 °C in neat OF. In the study carried out by Patteet et al. [22], it was reported that quetiapine remained stable for 7 days when stored at 4 °C.

Moreover, a 50% loss is observed after 44 days of DSS storage for CPZ, CLZ, and QTP, while LVP, CYA, and HAL do not present a 50% deviation after 146 days of study; these last three antipsychotics are considered more stable under the optimized conditions. It is important to consider that in the study of Caramelo et al. [1], the DSS samples were stored at room temperature. In the present work, the optimized conditions included ascorbic acid as preservative and storage in a refrigeration environment. However, according to the pareto charts in Figure 1, the effect of the ascorbic acid on the stability of the target antipsychotics seems to surpass that of temperature. It is possible to see that the bar of parameter C (preservative) is larger than that of parameter A (temperature) for most analytes.

## 3. Materials and Methods

### 3.1. Reagents and Standards

Standard solutions of chlorpromazine, haloperidol, and clozapine were purchased from LGC Promochem (Barcelona, Spain). Promazine, levomepromazine, and cyamemazine were purchased from Sigma Aldrich (Lisbon, Portugal). Quetiapine was kindly offered by AstraZeneca PLC (London, UK). All standards were acquired at 1 mg/mL. N-metil-N-(trimetilsilil) trifluoroacetamide (MSTFA) and trimethylchlorosilane (TMCS) were acquired from Macherey-Nagel (Düren, Germany). Deionized water was obtained from a Milli-Q System (Millipore, Billerica, MA, USA). Ascorbic acid was purchased from Fisher Scientific (UK), while sodium azide was purchased from Panreac Química SA (Barcelona, Spain), and sodium fluoride was provided by Sigma Aldrich (Sintra, Portugal). Whatman™ 903 protein saver cards were acquired from Solítica (Lisbon, Portugal).

A work solution with all target analytes was prepared by proper dilution of standard solutions with methanol to a final concentration of 10 µg/mL, except for haloperidol, which was diluted to 2 µg/mL. The Internal Standard (IS) Promazine was also diluted, with methanol, to a final concentration of 0.5 µg/mL. Promazine is an antipsychotic drug that is not commercially available in Portugal, so it is unlikely to be found in real patient samples. All the solutions were stored in the absence of light at 4 °C.

### 3.2. Biological Specimens

OF samples used in the present work were collected via the spitting method from drug-free donors of the laboratory staff and stored at 4 °C. A pool was prepared with OF from at least 5 different individuals. Before DSS application, OF was centrifuged at 3500 rpm for 5 min.

### 3.3. Gas Chromatographic and Mass Spectrometric Conditions

Chromatographic analysis was performed according to the method described by Caramelo et al. [1]. Thus, a gas chromatography system HP 7890A with a triple quadrupole detector (7000B), both from Agilent technologies (Waldbronn, Germany), a MPS2 autosampler and a PTV-injector from Gerstel (Mülheim an der Ruhr, Germany), were used. Antipsychotic drug separation was achieved with a capillary column (30 m × 0.25 mm I.D., 0.25 µm film thickness) with 5% of phenylmethyl siloxane (HP-5MS) supplied by J & W Scientific (Folsom, CA, USA). 

The oven temperature started at 120 °C for 2 min, followed by an increase of 20 °C/min to 300 °C, and held for 14 min. The total run time was 25 min. Injector temperature was set at 250 °C and the injections were made in splitless mode. The 2 µL extract injected was analysed through 35 µA filament current and electron energy of 70 eV in the positive electron ionization mode. Nitrogen was used as collision gas, at a flow rate of 2.5 mL/min. Data were acquired in the multiple reaction monitoring (MRM) mode using the MassHunter WorkStation Acquisition Software rev.B.02.01 (Agilent Technologies). 

Table 4 shows the retention times, quantifier and qualifier transitions, collision energies, and dwell times selected for each analyte under study.

### 3.4. Sample Preparation

The OF samples were prepared according to previously developed studies [1]. Thus, these were spiked with 200 ng/mL of the antipsychotics clozapine, levomepromazine, cyamemazine, chlorpromazine, and quetiapine, and 40 ng/mL of haloperidol. After homogenization, 50 µL of these mixtures were applied in the WhatmanTM 903 protein saver card and left to dry for 1 or 7 days. The spots were then cut and transferred to glass tubes with 2 mL of acidified methanol (pH = 5) and 25 µL of IS work solution. The extraction procedure was carried out under agitation on a roller mixer for 5 min at room temperature, and then centrifuged for 15 min at 3500 rpm. The supernatant solution was evaporated to dryness under nitrogen stream at 35 °C, and then derivatized with 50 µL of MSTFA with 5% of TMCS in a microwave oven at 800 W for 2 min. Finally, a 2 µL aliquot of the extract was injected into the GC-MS/MS system.

### 3.5. Design of Experiments

The DOE is a statistic tool that allows the definition of an experimental procedure with different factors, in order to identify the effect of each one and interactions between factors on the response [23]. With this multivariate approach, it is possible to minimize the number of experiments, optimize study conditions, and obtain better results [23,24]. In the present work, DOE was applied to evaluate the response to different storage conditions, and to optimize the long-term stability of compounds in DSS cards. Preservative concentrations were chosen according to the literature [25]. For each of the preservatives, more specific norms were followed. Thus, ascorbic acid concentrations were based on the study developed by Nielsen et al. [26]; on the other hand, sodium fluoride concentrations were based on sampling recommendations described by the International Association of Forensic Toxicologists (TIAFT)—Committee on Systematic Toxicological Analysis [27]. With regard to sodium azide concentrations, the manufacturer’s recommendations were followed.

Table 5 shows the experimental design matrix in which four factors were evaluated at two levels (2^4^). The factors were: (i) storage temperature (25 °C or 4 °C); (ii) presence or absence of light; (iii) concentration of preservative (300 ng/mL and 600 ng/mL for ascorbic acid, 1% and 2% for sodium fluoride and 0.1% and 0.2% for sodium azide); and storage time (1 and 7 days). This matrix was performed using the MINITAB^®^ software, version 17.

### 3.6. Long-Term Stability Evaluation

For the long-term stability evaluation, DSS spiked samples were stored at the DOE optimal conditions for 3, 7, 14, 21, 28, 218 44, 117, and 146 days. The response was measured by the relative peak areas (converted to concentration by a freshly prepared calibration curve) and compared to those obtained with DSS samples also freshly prepared. All freshly pre-pared samples involved a 24 h drying time period, corresponding to the minimum time period in our long-term stability evaluation, which was 1 day. A percentage of 100% was established for the concentration of the target analyte at day 1. 

## 4. Conclusions

The present work had the purpose of studying the stability of six antipsychotic drugs (chlorpromazine, levomepromazine, cyamemazine, clozapine, haloperidol, and quetiapine) in OF samples using different preservatives (ascorbic acid, sodium fluoride, and sodium azide) and different storage conditions. The DSS was used as sample storage and pre-treatment procedure, and a DOE was used for the optimization of the storage conditions. 

After analysing the results obtained by DOE, it was possible to conclude that the best storage conditions for the analytes under study would be with 300 ng/mL of ascorbic acid and storage of the DSS at 4 °C in the absence of light. However, preservative addition is only significant to the responses of clozapine, haloperidol, and quetiapine. Hence, it was possible to conclude that chlorpromazine and quetiapine remain stable for 14 days, levomepromazine was stable for 44 days, cyamemazine had a good stability over the entire monitored period (146 days), and clozapine and haloperidol were stable for 28 days.

This is the first study that determines the stability of the antipsychotic drugs chlorpromazine, levomepromazine, cyamemazine, clozapine, haloperidol, and quetiapine on OF samples applied to DSS using GC-MS/MS. The use of ascorbic acid as a preservative in OF samples applied on DSS and stored at 4 °C in the absence of light proved to be a good approach to improve these analytes stability in this matrix and allow an accurate long-term determination.

## Figures and Tables

**Table 1:** Response of all analytes between first and seventh days of storage at conditions A, B, C, and D.

Conditions	Chlorpromazine	Levomepromazine	Cyamemazine	Clozapine	Haloperidol	Quetiapine
A	0.055	1.003	0.036	0.043	0.140	0.059
B	0.094 *	1.784 *	0.053 *	0.060 *	0.086	0.084
C	0.074	1.312	0.047	0.058	0.171 *	0.110 *
D	0.052	1.092	0.039	0.035	0.160	0.079

A—Storage at 4 °C in presence of light; B—Storage at 4 °C in absence of light; C—storage at 25 °C in absence of light; D—storage at 25 °C in presence of light; *—better response.

**Table 2:** Response of analytes with and without preservative after storage for 1 and 7 days at 4 °C in the absence of light.

	Days	Chlorpromazine	Levomepromazine	Cyamemazine	Clozapine	Haloperidol	Quetiapine
Without preservative	1	0.078	1.687	0.043	0.061	0.101	0.095
	7	0.086	1.610	0.056	0.043	0.050	0.051
With preservative	1	0.075	1.427	0.059	0.108	0.197	0.312
	7	0.073	1.490	0.067	0.120	0.183	0.289

**Table 3:** Results of stability of antipsychotic drugs chlorpromazine, levomepromazine, cyamemazine, clozapine, haloperidol, and quetiapine over 146 days (n = 3).

Analytes	Days	Concentration (ng/mL)	S.D.	CV (%)	Relative Loss (%)
**Chlorpromazine**	1	197.2	19.7	10	
	3	199.8	40.0	20	0
	7	191.9	13.4	7	−4
	14	186.6	26.1	14	−6
	21	118.3	17.7	15	−41
	28	131.4	21.0	16	−34
	44	84.1	15.1	18	−57
	117	86.8	6.1	7	−56
	146	86.8	15.6	18	−56
**Levomepromazine**	1	206.4	26.8	13	
	3	226.9	18.2	8	10
	7	215.5	19.4	9	4
	14	172.4	20.7	12	−16
	21	165.9	23.2	14	−20
	28	171.7	22.3	13	−17
	44	166.3	33.3	20	−19
	117	130.5	7.8	6	−37
	146	136.5	10.9	8	−34
**Cyamemazine**	1	199.3	21.9	11	
	3	206.1	20.6	10	4
	7	226.4	18.1	8	13
	14	179.1	14.3	8	−9
	21	165.5	8.3	5	−16
	28	185.8	35.3	19	−6
	44	165.5	26.5	16	−17
	117	168.9	11.8	7	−15
	146	162.2	17.8	11	−18
**Clozapine**	1	203.3	8.1	4	
	3	207.0	20.7	10	2
	7	225.8	9.0	4	11
	14	178.8	26.8	15	−12
	21	169.4	28.8	17	−17
	28	167.5	28.5	17	−18
	44	97.9	16.6	17	−52
	117	67.8	12.2	18	−67
	146	80.9	8.1	10	−61
**Haloperidol**	1	42.8	4.7	11	
	3	35.9	6.5	18	−16
	7	39.8	4.8	12	−7
	14	40.0	2.0	5	−6
	21	34.6	1.4	4	−19
	28	34.1	2.4	7	−20
	44	26.5	3.7	14	−38
	117	29.6	5.0	17	−31
	146	22.2	1.6	7	−48
**Quetiapine**	1	212.0	17.0	8	
	3	200.4	38.1	19	−6
	7	196.3	31.4	16	−7
	14	174.6	24.4	14	−18
	21	114.1	19.4	17	−46
	28	124.3	18.7	15	−41
	44	84.2	15.2	18	−60
	117	89.7	17.0	19	−58
	146	85.6	16.3	19	−60

S.D.: standard deviation; CV: coefficient of variation.

**Table 4:** Retention times, selected transitions, collision energies, and dwell times for the identification of each analyte under study.

Analyte	Retention Time	Transitions (*m*/*z*)	Collision Energy (eV)	Dwell Time (µs)
Promazine *	11.12	283.1–238.2	15	50
**Chlorpromazine**	11.90	317.1–233.1 317.1–272.1	15 20	50 50
**Levomepromazine**	11.97	227.7–185.1 184.5–141.1	20 15	50 50
**Cyamemazine**	12.36	321.9–278.4 321.9–100.0	10 10	50 50
**Clozapine**	14.24	325.6–243.0 325.6 -270.3	10 20	50 50
**Haloperidol**	14.91	297.9–297.3 297.9–73.3	5 20	50 50
**Quetiapine**	18.90	209.3 -139.0 209.3–183.3	20 15	50 50

* Internal standard; Underlined transitions correspond to quantifier transitions.

**Table 5:** Experimental design matrix with four factors at two levels (2^4^).

Run Order	Temperature	Light	Preservative Concentration	Time
**1**	4 °C	Presence	Low	1 Day
**2**	4 °C	Presence	Low	7 Days
**3**	4 °C	Presence	High	7 Days
**4**	4 °C	Absence	Low	1 Day
**5**	25 °C	Presence	High	7 Days
**6**	25 °C	Absence	Low	7 Days
**7**	25 °C	Absence	High	7 Days
**8**	25 °C	Absence	Low	1 Day
**9**	4 °C	Absence	High	7 Days
**10**	25 °C	Absence	High	1 Day
**11**	25 °C	Presence	Low	7 Days
**12**	4 °C	Absence	Low	7 Days
**13**	25 °C	Presence	High	1 Day
**14**	4 °C	Presence	High	1 Day
**15**	25 °C	Presence	Low	1 Day
**16**	4 °C	Absence	High	1 Day

Each line of the matrix represents an experiment.

## Data Availability

The data presented in this study are available on request from the corresponding authors.

## Sample Availability

Not available.
